# Impact of treatment strategy and physical performance on future knee-related self-efficacy in individuals with ACL injury

**DOI:** 10.1186/s12891-018-1973-2

**Published:** 2018-02-13

**Authors:** Vala Flosadottir, Richard Frobell, Ewa M. Roos, Eva Ageberg

**Affiliations:** 1 Department of Health Sciences, Lund University, Box 157, 221 00 Lund, Sweden; 2 Department of Clinical Sciences, Lund University, Lund, Sweden; 3 Department of Sports Science and Clinical Biomechanics, Musculoskeletal Function and Physiotherapy, University of Southern Denmark, Odense, Denmark

**Keywords:** Knee, Patient outcome assessment, Performance-based measures, Psychological factors

## Abstract

**Background:**

In people with anterior cruciate ligament (ACL) injury, high self-efficacy facilitates recovery, indicated by improved muscle function, reduced knee symptoms and increased physical activity. Impact of treatment on future self-efficacy is however not well investigated. The aims of the study were to 1) investigate knee-related self-efficacy 6 years after acute ACL injury in patients treated with exercise therapy alone or in combination with either early or the option of delayed ACL reconstruction (ACLR), and 2) to investigate associations between single-leg physical performance at various time points after ACL injury and knee self-efficacy at 6 years after injury.

**Methods:**

Participants (*n* = 121) originated from the KANON-study (ISRCTN84752559), a treatment RCT including active adults with acute ACL injury treated with structured exercise therapy combined with early or the option of delayed ACLR. In this ancillary study, participants with knee self-efficacy data at 6 years (*n* = 89) were analyzed as treated; exercise therapy alone (*n* = 20), exercise therapy plus early ACLR (*n* = 46), and exercise therapy plus delayed ACLR (*n* = 23). The participants performed physical performance tests (hop, strength and balance) at the end the of exercise therapy (mean 10 (SD 6) months), and at 5 years, and rated their knee self-efficacy using Knee Self-Efficacy Scale (K-SES) questionnaire (0 to 10, worst to best) at 6 years.

**Results:**

Median K-SES score for the total group (*n* = 89) was 7.8 (IQR 5.9–9.0). There were no differences between treatment groups in K-SES scores at 6 years nor in physical performance at any time point (*p* ≥ 0.097). Worse knee flexion strength LSI (r_sp_ = 0.341, *p* = 0.042) at the end of the exercise therapy, and worse LSI for single-leg hop test (r_sp_ = 0.310, *p* = 0.005) at the end of the exercise therapy and at 5 years, correlated moderately with worse knee-related self-efficacy at 6 years. Low associations were observed between the remaining physical performance tests and K-SES scores (r_sp_ ≤ 0.265, *p* ≥ 0.045).

**Conclusion:**

Knee-related self-efficacy at 6 years after ACL injury did not differ between those treated with ACLR, performed early or as a delayed procedure, or exercise therapy alone. Good physical performance at the end of the exercise therapy, and at 5 years, appears to have a positive, yet small, impact on future knee-related self-efficacy.

## Background

In recent years, there has been increased focus on psychosocial factors and their association to recovery and return to sport (RTS) after anterior cruciate ligament (ACL) injury [1, 2]. The expectations of good recovery of knee function and RTS are high among individuals with ACL injury [3, 4]. However, despite treatment, with or without surgical reconstruction, impairments in patient-reported outcome measures (PROMs) [5, 6] and in physical performance often persist [7]. Only 81% return to any sport and 55% return to competitive level sports following an ACL injury or anterior cruciate ligament reconstruction (ACLR) [8].

The way in which a person reacts to an sport injury, i.e., the psychosocial response, includes cognitive, affective and behavioral aspects [9]. Negative psychological responses following an ACL injury or ACLR include pain and anxiety response, depression, and loss of athletic identity [10–12]. Positive psychological responses, on the other hand, include high motivation, high confidence and low fear of reinjury [1, 2] in addition to high self-efficacy [13]. These positive psychological responses interact both with each other and with the outcomes of the treatment [9]. In those with ACL injury, studies show that positive psychological responses, including high self-efficacy (cognitive response), facilitate recovery, in terms of improved muscle function, reduced knee symptoms and increased physical activity and that these are associated with higher return to pre-injury level of sports [1, 2]. However, we are not aware of any studies with the reverse design that evaluate whether modifiable treatment factors influence future self-efficacy.

Self-efficacy has been defined as the perception of one’s capability to perform a specific task, irrespective of whether one actually does or can perform it [14]. The level of self-efficacy can influence one’s initiative for action, level of effort and resilience to setbacks [14]. Previous experience of failure and success, including one’s own and through observation of others, can conversely effect one’s self-efficacy. [14] Commonly used measures evaluating knee-related self-efficacy after ACL injury, are the ACL-Return to Sports after Injury scale (ACL-RSI) [15], the Modified Self-Efficacy for Rehabilitation Outcome Scale (SER) [16], knee confidence with the single item Q3 from the Knee injury and Osteoarthritis Outcome Score subscale quality of life (KOOS item Q3) [17], and the Knee Self-Efficacy Scale (K-SES).

Low knee-related self-efficacy, in terms of low K-SES scores, has been reported in the early stages of treatment after ACL injury or reconstruction but is suggested to improve during the course of rehabilitation [18, 19]. One reason for this improvement in knee-related self-efficacy could be associated with decreased knee-related symptoms and the improvements in physical performance that are achieved during rehabilitation, however, to our knowledge this has not been studied. Another potential contributing factor for achieving high self-efficacy may be the treatment strategy, i.e., exercise therapy plus ACLR or exercise therapy alone. Thomeé et al. [20] investigated the role of knee-related self-efficacy in ACL-injured patients, undergoing exercise therapy with or without ACLR, and concluded that self-efficacy increased during the first year after injury and ACLR. The influence of self-reported knee function, age, gender and physical activity on the patients’ perceived knee self-efficacy was investigated [20]. However, the possible impact of different treatment strategies on future knee-related self-efficacy following ACL injury has not been reported.

Therefore, the aims of this study were to 1) report knee-related self-efficacy 6 years after acute ACL injury in patients treated with exercise therapy alone or in combination with either early or the option of delayed ACLR, and to 2) investigate associations between objectively measured single-leg physical performance at various time points after ACL injury and self-reported knee self-efficacy at 6 years after injury.

## Methods

### Participants

The participants in the present study originate from the Knee Anterior Cruciate Ligament, Nonsurgical versus Surgical Treatment (KANON) study (ISRCTN84752559) [5], a randomized controlled trial including 121 physically active adults with an acute ACL injury to a previously un-injured knee. The KANON-trial compared two treatments strategies: structured exercise therapy combined with an early ACLR (*n* = 62) or exercise therapy with the option of a delayed ACLR performed if needed (*n* = 59) [5]. The exercise therapy program was consistent with consensus in the literature [21]. At 2 and 5 years, there were no differences between the groups as randomized or as treated in knee-related outcomes, health status, return to preinjury activity level [5], or knee osteoarthritis (OA) frequency [6].

**Fig. 1:**
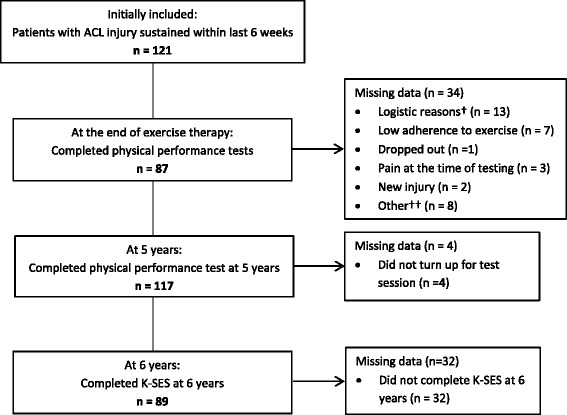
Flow chart of participants and follow-ups with physical performance tests and the Knee Self-Efficacy Scale (K-SES). †Long-distance relocation or transferal to a physical therapist (PT) not involved in the study. ††Pregnancy (*n* = 1), disc herniation (n = 1), advised against performing test by PT (*n* = 3), missing test protocols (n = 3). Participants with K-SES data and physical performance test data were included in the analyses

In this ancillary study all participants with available results on the K-SES (*n* = 89, Table 1) were included and analyzed according to treatment actually received: exercise therapy alone (ACL-D, *n* = 20), exercise therapy plus early reconstruction (ACL-R *n* = 46), and exercise therapy plus delayed reconstruction (ACL-X *n* = 23). Of the included participants, 62 performed the physical performance tests at the end of the exercise therapy period (mean 10 (SD 6) months after injury) [22] and 85 participants performed the physical performance test at 5 years after ACL injury; knee related self-efficacy was self-reported by mail at 6 years (Fig. 1). There were no differences in the physical performance tests between the groups as treated (ACL-D, ACL-R, ACL-X). The KANON study was approved by the Research Ethics Committee at Lund University, Sweden (LU 535–01) and all participants gave their written informed consent.

**Table 1:** Baseline characteristics of the study participants (*n* = 89)

	ACL-D (*n* = 20)	ACL-R (*n* = 46)	ACL-X (*n* = 23)	*p*-value
Age (y), mean (SD)	25.7 (4.8)	26.7 (5.4)	25.7 (4.8)	0.673
Female gender, n (%)	7 (35)	11 (24)	7 (30)	0.672
BMI (kg/m^2^), mean (SD)*	23.4 (2.4)	24.2 (3.3)	23.4 (2.0)	0.789
Tegner activity score, median (IQR)	9 (7–9)	8.5 (7–9)	8 (7–9)	0.934
Participating in sports when injured, n (%)	19 (95)	46 (100)	22 (96)	0.331
Right knee injured, n (%)	12 (60)	25 (54)	12 (52)	0.868
MRI-findings				
Total ACL-rupture, n (%)	19 (95)	46 (100)	23 (100)	0.175
Meniscal injury, n (%)**	12 (60)	32 (70)	9 (39)	0.052
Autograft type				
Patella tendon (%)		17 (37)	11 (48)	0.386
Hamstring tendon, n (%)		29 (63)	12 (52)	0.386
Supervised exercise therapy sessions^a^, mean (SD)	34 (23.2)	67 (32.3)	70 (38.1)	0.000

*n = 87, **n = 88, ^a^Number of supervised session until 2 yrs. follow-up

*SD* standard deviation, *IQR* inter-quartile range, *BMI* body mass index, *MRI* magnetic resonance imaging, *ACL* anterior cruciate ligament, *ACL-D* exercise only, *ACL-R* exercise plus early reconstruction, *ACL-X* exercise plus delayed reconstruction

### Physical performance

Physical performance was assessed at two time points; at the end of the exercise therapy and at 5 years follow-up. Seven single-leg physical performance tests, as described in Ericsson et al. [22] were assessed at the end of the exercise therapy when all goals of the exercise protocol were considered to be met [22]. The tests included the single-leg hop for distance, vertical hop, square hop, single-leg rise, single-leg balance test, knee extension strength, and knee flexion strength (Table 2). At mean 5 (SD 0.5) years after injury, performance was only assessed by the single-leg hop test for distance. The change in single-leg hop performance between the end of the exercise therapy and at 5 years was also calculated.

**Table 2:** Single-leg physical performance at the end of the exercise therapy at 10 months and at 5 years after ACL injury or reconstruction (*n* = 33–85) ^a^

	ACL-D *n* = 7–18	ACL-R *n* = 16–46	ACL-X *n* = 10–21	*p*-value
At the end of the exercise therapy, at mean 10 months (SD 6)				
Single-leg hop LSI, n = 62	101.4 (5.7)	99.8 (10.5)	100.5 (4.0)	0.588
Vertical hop LSI, n = 33	101.1 (9.4)	91.8 (27.3)	101.1 (9.4)	0.144
Square hop LSI, n = 61	106.7 (12.2)	103.4 (11.7)	98.9 (16.8)	0.563
Single-leg rise LSI, *n* = 61	99.5 (5.9)	97.6 (6.5)	98.1 (5.2)	0.264
Single-leg balance LSI, n = 61	116.8 (50.0)	130.6 (67.3)	126.3 (74.8)	0.863
Knee extension, LSI (*n* = 49)	98.2 (8.3)	97.8 (9.3)	100.1 (5.1)	0.484
Knee flexion, LSI (n = 33)	93.7 (11.3)	98.4 (8.9)	107.7 (16.7)	0.097
At mean 5 years (SD 1)				
Single-leg hop LSI, *n* = 85	97.5 (11.5)	95.2 (12.0)	94.8 (12.7)	0.586
Change between 10 months and 5 years				
Single-leg hop LSI, n = 61	−4.2 (13.9)	−1.8 (12.6)	6.6 (13.3)	0.556

Values are the mean (SD)

*LSI* Limb Symmetry Index, *ACL-D* exercise only, *ACL-R* exercise plus early reconstruction, *ACL-X* exercise plus delayed reconstruction

^a^Missing values due to equipment problems or patients declining to perform a test

The Limb Symmetry Index (LSI) was used for all analyses of physical performance. The LSI was calculated by dividing the result for the injured leg by that of the uninjured leg and multiplying by 100 [23].

Thirty-four participants (9 women, 25 men) did not complete the physical performance tests at the end of the exercise therapy (Fig. 1), and, were excluded from the correlation analyses. These participants did not differ from those who completed the physical performance tests with respect to age (*p* = 0.41), gender (*p* = 0.99), BMI (*p* = 0.24), activity level (*p* = 0.96), treatment randomized (*p* = 0.30), or number of supervised exercise therapy sessions (*p* = 0.32). Depending on data normality and type, the χ^2^ test or the Mann-Whitney test were used for the between-group comparisons, as appropriate.

### Knee-related self-efficacy

Perceived knee-related self-efficacy was self-reported at mean 6 (SD 1) years after injury using the K-SES. K-SES consists of four sections; A – daily activities, B – sports and leisure activities, C – knee function tasks and D – future knee function. The full K-SES includes 22 items and the individual rates the certainty about the capability of performing an activity on an 11-point Likert scale, ranging from 0 (not at all certain) to 10 (very certain). Item scores are summarized and divided by the number of items for each section (A-D), yielding a total K-SES score ranging from 0 to 10 [24]. Two subscores, K-SES Present and Future, are also calculated each with a range of 0 to 10 [24]. In sections A through C, the participants report their perception about their present capability of performing knee-related tasks, yielding the subscore K-SES Present. In section D, they report their perception about their future knee function capability, such as return to preinjury level sport and fear of reinjury (plus decreasing knee function if undergoing ACLR), yielding the subscore K-SES Future. [24] The K-SES has been assessed for the following measurement properties; internal consistency, test-retest reliability, face validity, content validity and construct validity [20, 24]. The K-SES is reliable and valid for measuring perceived self-efficacy in patients with an ACL injury [24].

### Statistical analysis

Data were analyzed on a post-hoc as-treated basis. The one-way ANOVA test was used for the comparison of age at baseline between the treatment groups, and, the Kruskal-Wallis test was used for between-group comparisons of the BMI, the performance tests and the K-SES scores in the three treatment groups. Spearman’s, and partial Spearman’s, rank-order correlation analyses were used to assess associations between physical performance and K-SES, controlling for age, gender and treatment. Correlation coefficients thresholds suggested by Cohen [25] were used as follows; ≥ 0.10 to 0.29 denote low association, ≥ 0.30 to 0.49 moderate association and coefficients ≥0.50 large association. *P*-values less than or equal to 0.05 were considered statistically significant. Statistical analyses where performed using IBM SPSS for Windows, version 23.0 (IBM corp., Armonk, NY, USA).

## Results

**Fig. 2:**
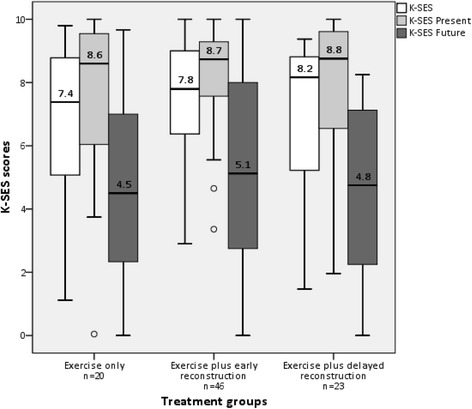
Distribution of Knee Self-Efficacy (K-SES) scores in the different treatment groups (total *n* = 89) at 6 years after injury. The bar in the box represents the median, the box represents the interquartile range (IQR) and the whiskers extend to the minimum or the maximum values within 1.5 IQR from the lower and the higher edges of the box. Circles represent cases with values between 1.5 and 3 IQR from the lower edge of the box

The median K-SES scores at 6 years for all participants (*n* = 89) were K-SES 7.8 (IQR 5.9–9.0), K-SES present 8.7 (IQR 6.8–9.6) and K-SES future 4.8 (IQR 2.5–7.5). There were no differences between the three treatment groups in K-SES scores 6 years after injury (*p* ≥ 0.501, Fig. 2).

There were no differences between treatment groups in single-leg physical performance at any time point (*p* ≥ 0.097, Table 2). Therefore, the correlation analyses were performed with data from all patients combined. Worse LSI for knee flexion strength (r_sp_ = 0.341, *p* = 0.042) at the end of the exercise therapy, as well as worse LSI on the single-leg hop test at 5 years (r_sp_ = 0.310, *p* = 0.005), correlated moderately with worse knee-related self-efficacy at 6 years after injury (Table 3). Low associations were observed between the remaining physical performance tests at the end of the exercise therapy and K-SES, ranging from r_sp_ = 0.148 (*p* = 0.264) for the LSI for the single-leg hop test to r_sp_ = 0.265 (*p* = 0.045) for the LSI in the square hop test.

**Table 3:** Spearman’s rank-order correlations (r_s_) and rank-order partial correlations (r_sp_) between physical performance at the end of the exercise therapy and at 5 years after ACL injury/ACLR and K-SES scores at 6 years

	K-SES (n = 33–85)			
	r_s_	p-value	r_sp_^a^	*p*-value
At the end of the exercise therapy, at 10 months				
Single-leg hop LSI	0.150	0.244	0.148	0.264
Vertical hop LSI	−0.142	0.431	−0.236	0.210
Square hop LSI	0.267	0.037	0.265	0.045
Single-leg rise LSI	0.163	0.209	0.190	0.153
Single-leg balance LSI	0.237	0.066	0.238	0.072
Knee extension LSI	0.130	0.375	0.167	0.267
Knee flexion LSI	0.286	0.078	**0.341**	0.042
At 5 years				
Single-leg hop LSI	0.298	0.006	**0.310**	0.005
Change between 10 months and 5 years				
Single-leg hop LSI	0.201	0.120	0.196	0.139

Bold numbers represent moderate correlations. Regular numbers represent low correlations. Correlation coefficients: ≥ 0.10 to 0.29 denote low association, ≥ 0.30 to 0.49 moderate association and coefficients ≥0.50 large association

*K-SES* Knee Self-Efficacy Scale, *LSI* Limb Symmetry Index

^a^Physical performance effect independent of age, gender and treatment

## Discussion

Knee-related self-efficacy at 6 years after acute ACL injury was not associated with treatment strategy, in terms of exercise therapy alone or combined with early or optional delayed ACLR. Worse LSI in knee flexion strength at the end of the exercise therapy and worse LSI in the single-leg hop test for distance, at 5 years, were moderately associated with worse knee-related self-efficacy at 6 years.

The K-SES scores (median 7.4–8.2) for our cohort were comparable, or better, than K-SES scores previously reported from other Swedish cohorts: mean 6.8 and 7.6 at 12 months after ACL injury and ACLR, respectively [20]; mean 7.3 at 12 months after ACL injury or ACLR [26]; and median 6.7 at 1–12 month after ACL injury and 3–12 months after ACLR [24].

In a previous study, similar levels of K-SES scores (mean 6.9–8.3), have been shown to be associated with greater likelihood of satisfaction with knee function, at mean 3 years after ACLR [27]. In the present study, the K-SES Present scores (perception about one’s present capability of performing knee-related tasks) can be considered to be high, whereas the K-SES Future scores (perception about one’s return to preinjury level sports and fear of reinjury plus decreasing knee function if undergoing ACLR) were remarkably low, for all three treatment groups. In contrast, previous studies report that the K-SES Future scores correspond to or are higher than the K-SES Present scores [18, 20, 24, 26]. One explanation for this could be that previous studies investigated knee-related self-efficacy within one month after ACL injury or before ACLR and up to 1 year after injury or ACLR [18, 20, 24, 26], whereas in the present study we assessed knee-related self-efficacy at 6 years after ACL injury or ACLR. The K-SES Future score refers to perceptions about return to preinjury level sports, fear of reinjury and decreasing knee function. Previous reports have shown, that less than 50% return to sports, at preinjury or competitive level, at 2–7 years after ACLR [28]. In the KANON-trial, only about 20% of the participants were active at their preinjury activity level or higher at 5 years after structured rehabilitation with or without the addition of ACLR [6]. Thus, it may be assumed that no more than 20% of participants in the present cohort were active at their preinjury activity level at 6 years. This may be reflected by the low K-SES Future scores presented in the present study. Fear of reinjury, despite the perception of good knee function, has been shown to be the most common hindrance for return to sports [29, 30]. This may explain the discrepancy between the high K-SES Present (perception of good knee function capability) and the low K-SES Future (uncertainty about return to preinjury level sports and reinjury) scores. In addition, the expectations and motivation of return to preinjury level sports and the perception of acceptable knee function may change over time. High, and potentially unrealistic, expectations of return to sports and knee function have been reported by patients prior to surgery for an ACL or a meniscus injury [3, 4, 31]. As in the current study, lower, and perhaps more realistic, expectations may be expressed several years later. Therefore, other potentially relevant explanations for the low K-SES Future scores may be natural changes, including increasing age or changes in social commitments. A longitudinal study is required to further investigate these potential explanations for change in future knee self-efficacy in individuals after ACL injury.

The lack of differences in K-SES scores between groups as treated (ACL-D, ACL-R and ACL-X) is in line with previous observations for various outcomes of the KANON-trial, in terms of self-reported outcomes [5] and osteoarthritis (OA) [5, 6] at 2 or 5 years, and muscle function [7] evaluated at 3 years after ACL injury. All participants followed the same exercise therapy protocol regardless of treatment strategy. The as treated group, receiving exercise therapy alone, progressed faster and needed fewer exercise sessions than the two groups who had early or delayed ACLR in addition to exercise therapy. The exercise program was goal-based and progression to a more strenuous level was allowed when the goals for range of motion, muscle function, and functional performance, respectively, were met [5]. As a result, the exercise program was based on functional goals rather than a pre-determined number of sessions. This approach was chosen to ensure that all treatment groups would achieve a similar and good level of physical function. Thus, the extensive exercise therapy program, rather than the early or the optional delayed ACLR, may be the common factor explaining our findings of similar K-SES scores across all treatment groups.

To our knowledge, our study is the first to investigate the influence of physical performance on knee-related self-efficacy after ACL injury. In contrast, previous studies have the reverse design, focusing on the influence of psychological responses on physical function and performance [32]. Higher knee-related self-efficacy, in terms of higher K-SES score, before ACLR has been reported to be associated with good physical performance (LSI ≥ 90 in single-leg hop and muscle power tests) and improved self-reported knee function (KOOS) at 1-year follow-up after ACLR [33]. Our results indicate that greater (better) LSI for knee flexion strength at the end of exercise therapy after ACL injury, correlated with better knee-related self-efficacy (higher K-SES scores) at 6 years. Although these associations were only moderate, our findings are supported by results from previous longitudinal studies, indicating that symmetry between legs in single-leg physical performance after exercise therapy, or within one year after ACL injury or ACLR, is one contributing factor for good future self-reported outcomes, such as, self-reported knee function and high return to pre-injury sports [34–36].

We observed that worse LSI in single-leg physical performance at 5 years correlated moderately with worse knee-related self-efficacy at 6 years. Longitudinal results have previously shown that worse single-leg physical performance after ACL injury or ACLR was associated with worse knee confidence, a construct similar to knee-related self-efficacy. Lower (worse) LSI for single-leg hop performance at 3 years after ACL injury has been shown to be associated with worse knee confidence (KOOS item Q3) at 5 years [37]. These moderate associations between single-leg physical performance and knee confidence found in a previous study [37] and in our present longitudinal study, suggest that good single-leg physical performance is one factor contributing to better psychological outcome after ACL injury or ACLR. Further studies are required to evaluate the relative contribution of physical performance, as well as other factors, that may play a role for the psychological response after injury.

The level of knee-related self-efficacy is important after ACL injury and surgery as it can influence one’s initiative for action, level of effort and resilience to setbacks. Surgical treatment in addition to exercise therapy after ACL injury does not appear to influence later knee-related self-efficacy. The findings of the current study show that after ACL injury, patients may benefit from training that targets symmetry between legs, specifically, in knee flexion muscle strength and in single-leg hop performance.

The main strength of the current study is that data originates from the KANON-trial, a high quality RCT. Another strength is that there were no differences between participants and non-participants in baseline characteristics. There are some limitations to the present ancillary study. The completion rates for the physical performance tests at the end of the exercise therapy were between 33% and 70% and only 74% of the participants of the KANON-trial completed the K-SES questionnaire at 6 years after injury. These low completion rates may be explained by equipment problems at the test sites, or participants declining to perform test at the end of the exercise therapy, and that the K-SES was sent out by mail instead of being filled out at a clinical visit at 6 years. However, the majority of the participants included in this study completed the physical performance test at 5 years (96%). Additional limitations are that several testers assessed the physical performance tests at the end of the exercise therapy [22]. However, all assessors were experienced and similar written instructions were used at all centers and for all tests. The use of LSI for the analyses of the physical performance tests may be considered a limitation. Despite the LSI being a common measure, it may misrepresent the performance of the injured leg [38]. Bilateral deficiencies in hop tests have been reported in individuals after ACLR [39]. Since the LSI represents the level of limb symmetry, a high (good) LSI may be the result of either a good, or poor, performance of both legs. Lastly, K-SES was only assessed at 6 years after injury, not allowing adjustment for baseline scores or analyses of change in K-SES scores.

## Conclusions

Knee-related self-efficacy at 6 years after an acute ACL injury did not differ between patients treated with ACL reconstruction, performed early or as a delayed procedure, or those treated with exercise therapy alone. At 6 years after injury, the self-efficacy for present knee function capability (K-SES subscore present) was higher than the self-efficacy for return to preinjury level sport and not being reinjured (K-SES subscore Future). Worse single-leg physical performance at the end of the exercise therapy period, and at 5 years, was weakly to moderately associated with worse knee self-efficacy at 6 years. Targeted training to improve the symmetry between legs in knee flexion muscle strength and in the single-leg hop for distance may have a positive, yet small, long-term impact on knee-related self-efficacy after ACL injury.

## Abbreviations

ACL: Anterior cruciate ligament
ACL-D: exercise therapy alone
ACLR: Anterior cruciate ligament reconstruction
ACL-R: exercise therapy plus early reconstruction
ACL-RSI: the ACL-Return to Sports after Injury scale
ACL-X: exercise therapy plus delayed reconstruction
BMI: Body mass index
IQR: Inter-quartile range
KANON: the Knee Anterior Cruciate Ligament, Nonsurgical versus Surgical Treatment
KOOS item Q3: the single item Q3 from the Knee injury and Osteoarthritis Outcome Score subscale quality of life
KOOS: the Knee injury and Osteoarthritis Outcome Score
K-SES: the Knee Self-Efficacy Scale
LSI: Limb symmetry index
MRI: Magnetic resonance imaging
OA: Osteoarthritis
PROMs: in patient-reported outcome measures
PT: Physical therapist
r_s_: Spearman’s rank-order correlations
r_sp_: Spearman’s rank-order partial correlations
RTS: Return to sports
SD: Standard deviation
SER: the Modified Self-Efficacy for Rehabilitation Outcome Scale
